# Modulation of cerebral blood flow and cognition by hyperthermia and hypoxia: An electroencephalographic event‐related potentials perspective

**DOI:** 10.1113/EP092671

**Published:** 2025-12-12

**Authors:** Hiroki Nakata, Shigehiko Ogoh, Manabu Shibasaki

**Affiliations:** ^1^ Faculty of Engineering Nara Women's University Nara Japan; ^2^ Department of Biomedical Engineering Toyo University Asagiri Japan

**Keywords:** event‐related potential, heat stress, hypoxia, P300

## Abstract

Cerebral blood flow (CBF) is essential for sustaining neuronal metabolism and cognitive performance; however, the precise relationship between perfusion and cognition remains unclear. Although ageing and disease are associated with progressive declines in CBF and cognitive impairment, the acute effects of altered CBF under environmental stressors have not been elucidated fully. The influence of environmental stress on cognitive function is likely to depend on the degree of stress (e.g., its intensity and duration). Therefore, it is necessary to carry out a systematic review of a large number of studies, and objective evidence is required to build a comprehensive dataset. This review summarizes research examining the effects of mild to moderate passive heat stress (an increase in core temperature of ∼1.0°C–1.5°C) and acute hypoxia on cognitive processing, as evaluated using electroencephalographic event‐related potentials (EEG‐ERPs), with the aim of facilitating future cross‐experimental comparisons. During mild or greater hyperthermia, CBF decreases owing to blood flow redistribution and hypocapnia‐induced by hyperventilation, whereas during hypoxia, CBF can either increase or decrease depending on the conditions (e.g., exposure time or intensity). To standardize comparisons, this review focuses on acute hypoxic exposures, during which CBF tends to decrease. Although it is undeniable that the content summarized here might be somewhat selective, it is hoped that this foundation will contribute to the future development of constructive and objective evaluations. Current evidence indicates that acute fluctuations in CBF are unlikely to predict cognitive outcomes. Rather, both heat and hypoxic stress appear to impair neural activity through mechanisms beyond perfusion alone.

## Abstract (highlights)

- What is the topic of this review?
  Consciousness is lost once cerebral blood flow (CBF) falls below a critical threshold. Both hyperthermia and hypoxia can lower CBF, partly owing to hyperventilation‐induced hypocapnia that subsequently reduces cerebral perfusion. Although severe levels of both might contribute directly to loss of consciousness, it is plausible in moderate conditions that factors such as elevated brain temperature or reduced oxygen availability in the brain play a more direct role in cognitive impairment.
- What advances does it highlight?
  We explore the possibility that reductions in CBF induced by these two types of environmental exposures might not be the primary cause of cognitive decline in moderate conditions.

## 1 INTRODUCTION

Cerebral blood flow (CBF) plays a fundamental role in maintaining optimal cognitive function by ensuring a continuous supply of oxygen and metabolic substrates to neurons and glial cells (Iadecola, 2017). Despite accounting for only ∼2% of the total body weight, the brain receives ∼15%–20% of cardiac output and consumes ∼20% of the oxygen supply at rest, reflecting its remarkedly high metabolic demand (Raichle & Gusnard, 2002). Ageing is closely associated with a progressive decline in cognitive abilities, often accompanied by alterations in CBF. Population‐based studies suggest that resting CBF begins to decrease from around the age of 30 years, at an approximate rate of 4 mL/min/year (de la Torre, 2012). These reductions can occur decades before the onset of overt cognitive impairment and are strongly associated with an increased risk of neurodegenerative diseases, including Alzheimer's disease (Iturria‐Medina et al., 2016; Li et al., 2025). Longitudinal data further indicate that individuals with lower baseline CBF are more likely to develop mild cognitive impairment or dementia over time (Wolters et al., 2017), supporting the vascular hypothesis of cognitive decline.

Beyond ageing, lifestyle interventions, particularly physical exercise, have been investigated as potential modulators of CBF and cognition. With advancing age, both global CBF and cerebral metabolic rate of oxygen (CMRO_2_) decline by ∼20%–25% from young adulthood (Leenders et al., 1990; Tarumi et al., 2014). This parallel reduction suggests that the decline in CBF might partly reflect reduced metabolic demand rather than vascular impairment alone, implying preserved neurovascular coupling in healthy ageing. Previous meta‐analyses (Ishihara et al., 2021; Lambourne & Tomporowski, 2010) have demonstrated that even a single session of aerobic and/or resistance training can transiently enhance cognitive function, with notable effects on executive function, attention and processing speed. These acute benefits have been observed across various age groups, including older adults, suggesting that exercise might serve as an accessible, non‐pharmacological strategy for prevention of dementia (Basso & Suzuki, 2017; Groot et al., 2016). Proposed mechanisms include acute increases in CBF and oxygen delivery (Ogoh & Ainslie, 2009), upregulation of neurotrophic factors, such as brain‐derived neurotrophic factor (Winter et al., 2007), modulation of neurotransmitter systems (McMorris, 2021) and enhancement of neurovascular coupling (Lucas et al., 2015). However, the relative contributions of these mechanisms remain unclear, and the optimal exercise modality, intensity and duration for maximizing cognitive benefits have yet to be established. An important unresolved question is whether the relationship between CBF and cognitive function is mediated by similar physiological mechanisms across different contexts, including resting environmental conditions, exercise and the ageing process. Although increased CBF is generally considered to enhance cognitive function, recent findings suggest that this relationship might not be strictly linear or causal in all circumstances (Ogoh, 2017). Conversely, does a decrease in CBF impair cognitive function? Clinically, it has been proposed that alterations in cerebral haemodynamics might represent one of the potential mechanisms underlying cognitive decline (Toth et al., 2017).

Exposure to extremely hot or high‐altitude environments often results in slower cognitive processing and impaired judgment, because both hyperthermia and hypoxia can reduce CBF. Electroencephalographic event‐related potentials (EEG‐ERPs) offer a more objective means of assessing cognitive function (Nakata et al., 2021, 2017; Shibasaki et al., 2016, 2017). ERPs reflect higher‐order cognitive processes, such as attention and memory updating, with component amplitudes indicating the magnitude of neurocognitive processing (Duncan et al., 2009; Kok, 2001). In this review, we discuss the effects of reduced CBF on cognitive function in either hyperthermic or hypoxic conditions, presented in two sections: one focusing on hyperthermia and the other on hypoxia. The first section examines this relationship in mild passive heat stress, conditions known to reduce cerebral perfusion whilst redistributing blood flow to the skin for thermoregulation (Ogoh et al., 2013). The second section simulates high‐altitude conditions by manipulating inspired oxygen levels. It is important to note that many factors influencing cognition at high altitude are not replicated simply by changing the fraction of inspired oxygen (e.g., poor sleep or dehydration). Therefore, the experimental data discussed in this review were obtained during normobaric hypoxia. Unlike high‐altitude field studies, where factors such as sleep disturbance or dehydration can confound cognitive outcomes (Hayashi et al., 2005; Virués‐Ortega et al., 2004), normobaric conditions allow the effects of hypoxia per se to be isolated.

## 2 EVALUATION OF COGNITIVE FUNCTION

According to the definition provided by the Japanese Society of Psychiatry and Neurology, cognitive function is broadly classified into six categories: (1) complex attention; (2) executive function; (3) learning and memory; (4) language; (5) perceptual–motor function; and (6) social cognition (Takahashi et al., 2014). Cognition is based on the brain receiving information through sensory organs associated with the five senses (vision, hearing, touch, smell and taste), regarding both external stimuli and the internal state of the body. This sensory information is processed through perception, which is shaped by learning, knowledge and experiences. Subsequently, motor commands are transmitted from the brain to skeletal muscles, resulting in motor behaviour. Even simple actions, such as walking, require multiple cognitive functions. For example, in a crowded city, individuals avoid collisions by estimating the walking speed of oncoming pedestrians, or, when encountering someone moving slowly ahead, quickly calculate spatial and temporal gaps to adjust their own speed and overtake. Likewise, everyday activities, such as shopping, driving or using public transport, are dependent on cognitive function. In addition, environmental conditions can act as disturbing factors. Cognitive functions can deteriorate in various environmental conditions, leading to physical and mental health problems and impairments in daily life. Therefore, in modern society, it is essential to implement strategies and initiatives aimed at maintaining cognitive function and preventing its decline in diverse environmental conditions.

To quantify and objectify cognitive function, various neurophysiological methods, such as electroencephalography (EEG), magnetoencephalography (MEG) and transcranial magnetic stimulation (TMS), in addition to neuroimaging techniques, including functional magnetic resonance imaging (fMRI), functional near‐infrared spectroscopy (fNIRS) and positron emission tomography (PET), have long been used. In this review, we introduce our experiments using EEG‐based ERPs. EEG is based on an electrophysiological technique and characterized by its high temporal resolution, of the order of milliseconds. Unlike other measurement devices, EEG imposes relatively few experimental constraints, making it widely applicable in studies involving children, including infants. When neurons in the brain become active, they generate electrical currents; EEG detects and records these currents from the scalp surface. However, a notable limitation of EEG is its low spatial resolution. This is attributable to the presence of multiple intervening layers with markedly different conductivities, such as CSF, dura mater, skull, skin and air, between the brain and the scalp, which significantly affect the recorded signals and make it challenging to determine the exact source of brain activity accurately.

P300 (P3) component, obtained by time‐locked averaging the EEG, have been used to evaluate higher cognitive functioning that involves selective attention, expectancy and memory updating (Duncan et al., 2009). The amplitude of P300 component reflects the intensity of neurocognitive processing (Kok, 2001). Larger amplitudes indicate more neural excitability and/or neuronal circuits related to endogenous and exogenous components of the amount of attentional resources (Duncan et al., 2009; Mizukami et al., 2019). However, smaller or decreased amplitudes have been observed in untrained, aged or exhausted individuals (Shibasaki et al., 2019, 2016; van Dinteren et al., 2014; Yamashiro et al., 2015).

## 3 MILD HEAT STRESS CONDITIONS

Passive heat stress causes multiple physiological responses, many of which are directed towards heat dissipation to maintain thermal homeostasis. In particular, heat stress‐induced hyperthermia, with an increase in core temperature of ∼1.0°C or more, reduces cerebral blood velocity and cerebral vascular conductance (Low et al., 2008; Wilson et al., 2006, 2002). This reduction results from a redistribution of blood towards the cutaneous circulation and hyperventilation‐induced hypocapnia (Figure 1). In addition, the redistribution of blood flow to the skin decreases central blood volume and venous return, leading to a decrease in mean arterial pressure and, consequently, cerebral perfusion pressure (Bain et al., 2014). Although restoration of arterial CO_2_ through hypercapnic gas inhalation can partly recover CBF, it does not completely normalize it, indicating that reductions in central blood volume and systemic pressure, rather than hypocapnia alone, play a major role in limiting cerebral perfusion during heat stress (Brothers et al., 2009). The brain has a high metabolic rate and, therefore, requires continuous blood flow to supply oxygen and nutrients whilst removing heat. Consequently, the combination of increased CMRO_2_ owing to cognitive tasks and restricted CBF owing to hyperthermia further elevates brain temperature. Therefore, hyperthermia can exert marked effects on brain function and integrity. Empirically, exposure to hot environments often leads to slowed thinking or impaired judgment. Thus, both reduced cerebral perfusion and increased brain temperature can contribute to impairments in neural network function and cognitive processing.

**FIGURE 1:**
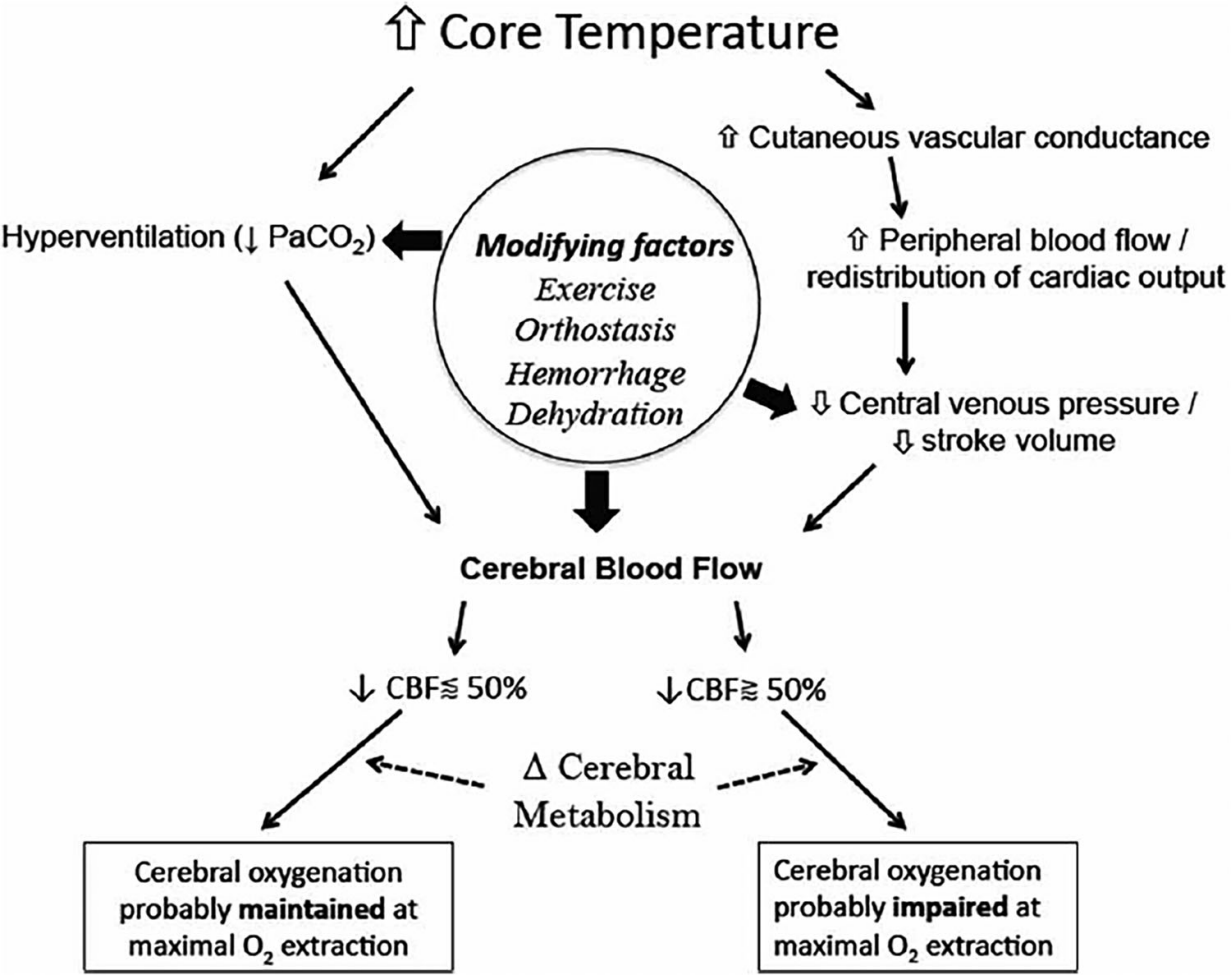
Simplified schematic of the mechanisms and modifying factors involved with reductions in cerebral blood flow and cerebral oxygenation during whole‐body hyperthermia. Abbreviations: CBF, cerebral blood flow; $P_{\mathrm{aC}O_{2}}$, arterial partial pressure of carbon dioxide. Reproduced from Bain et al. (2014).

Previously, many research groups indicated that hyperthermia impairs cognitive function, but others suggested that the effects might be minimal or task dependent; simple tasks might be unaffected by hyperthermia, whereas complex tasks show impairment (Gaoua et al., 2018; Hancock & Vasmatzidis, 2003). However, differences in methodology across studies have made it difficult to determine whether hyperthermia adversely affects cognitive function (Amos et al., 2000; Bell et al., 1964; Cian et al., 2001; Hancock, 1982; Hocking et al., 2001; Nunneley et al., 1979, 1982). The degree of hyperthermia might also influence the extent of cognitive impairment. Therefore, we first classify heat stress based on the level of core temperature elevation, as proposed by Bain et al. (2015), with mild, moderate and severe heat stress corresponding to increases in core temperature of ≤1.0°C, 1.0–1.5°C and ≥1.5°C, respectively. In this short review, we focus on mild to moderate elevations in core temperature, which have been primarily examined in previous studies investigating cognitive function during hyperthermia. The EEG recorded during cognitive tasks in hot environments might provide valuable insight into the underlying neural mechanisms. Previous studies primarily undertook frequency analysis of EEG signals. In particular, an increase in frontal midline theta power was observed during working memory and mental arithmetic tasks during heat stress (Gevins et al., 1997; Mizuhara et al., 2004). We hypothesized that hyperthermia might impair cognitive function even in simple tasks. Using EEG‐ERPs, we found a reduced peak amplitude of P300 during heat stress, suggesting impaired cognitive processing (Nakata et al., 2021; Shibasaki et al., 2016, 2017). In those studies, three different modalities (auditory, somatosensory and visual stimuli) and three different simple cognitive protocols (oddball, go/no‐go and flanker tasks) were adopted, and all showed similar reductions in P300 amplitudes during mild heat stress.

One approach to mitigate reductions in CBF during hyperthermia is the inhalation of hypercapnic gas (Brothers et al., 2009; Tsuji et al., 2018). However, it has been reported that CBF does not return to pre‐heat‐stress levels even with hypercapnic gas inhalation. Also, heat stress elicits feelings of displeasure, which might also contribute to impaired cognitive function. Notably, cold or cool stimuli applied to the body surface are perceived as comfortable and can enhance alertness when the body temperature is elevated (Nakamura et al., 2008). Indeed, marked increases in core and/or skin temperature have been demonstrated to reduce performance in complex cognitive tasks (Hancock, 1982; Simmons et al., 2008). Given these natural behaviours and the fact that heat stress increases skin blood flow in the face and head to facilitate heat dissipation (Ogoh et al., 2013), facial and head cooling have been hypothesized to improve subjective comfort and cerebral perfusion during heat stress. However, the effects of such cooling on cognitive function and/or cerebral perfusion have not been investigated thoroughly.

In our previous study (Shibasaki et al., 2017), we designed an experiment to dissociate the effects of reduced cerebral perfusion and increased brain temperature on cognitive function. To this end, core temperature was maintained whilst applying facial and head cooling, allowing the assessment of thermal comfort and cerebral perfusion independently of brain temperature. Participants completed four sessions of the somatosensory go/no‐go task: rest (first session: Rest); during heat stress (second session: HS); during heat stress with facial and head cooling (third session: FHC); and during whole‐body cooling after heat stress (fourth session: WBC). Figure 2 shows grand‐averaged ERP waveforms. The amplitudes of both go‐P300 and no‐go‐P300 were clearly smaller during HS and FHC than at Rest. *Post hoc* testing demonstrated that the amplitude of go‐P300 was significantly smaller during HS and FHC than Rest, and that of no‐go‐P300 was significantly smaller during HS, FHC and WBC than Rest (Figure 3). Thus, executive and inhibitory processing, as assessed by ERPs, did not recover during FHC. This finding suggests that restricted cerebral perfusion is not the sole factor underlying impaired neural network function and reduced cognitive processing.

**FIGURE 2:**
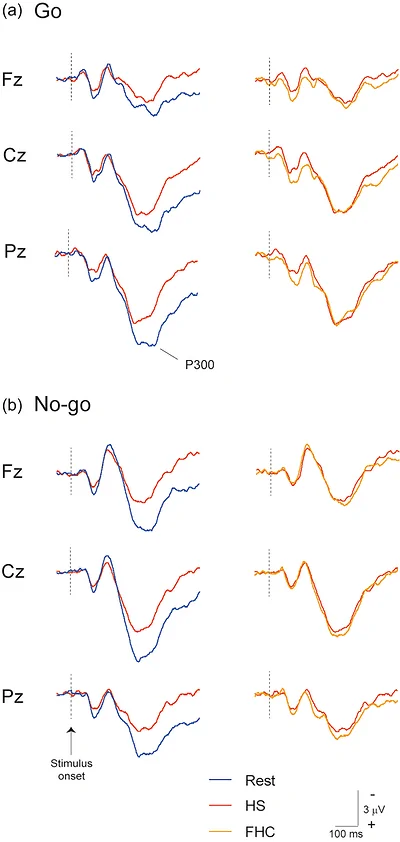
Grand‐averaged event‐related potential (ERP) waveforms at three electrode sites (Fz, Cz and Pz) in go/no‐go trials for each session. Amplitudes of the P300 component in both trials were clearly reduced during heat stress (HS) and remained reduced during face/head cooling (FHC). Adapted from Shibaskai et al. (2017), with modifications.

**FIGURE 3:**
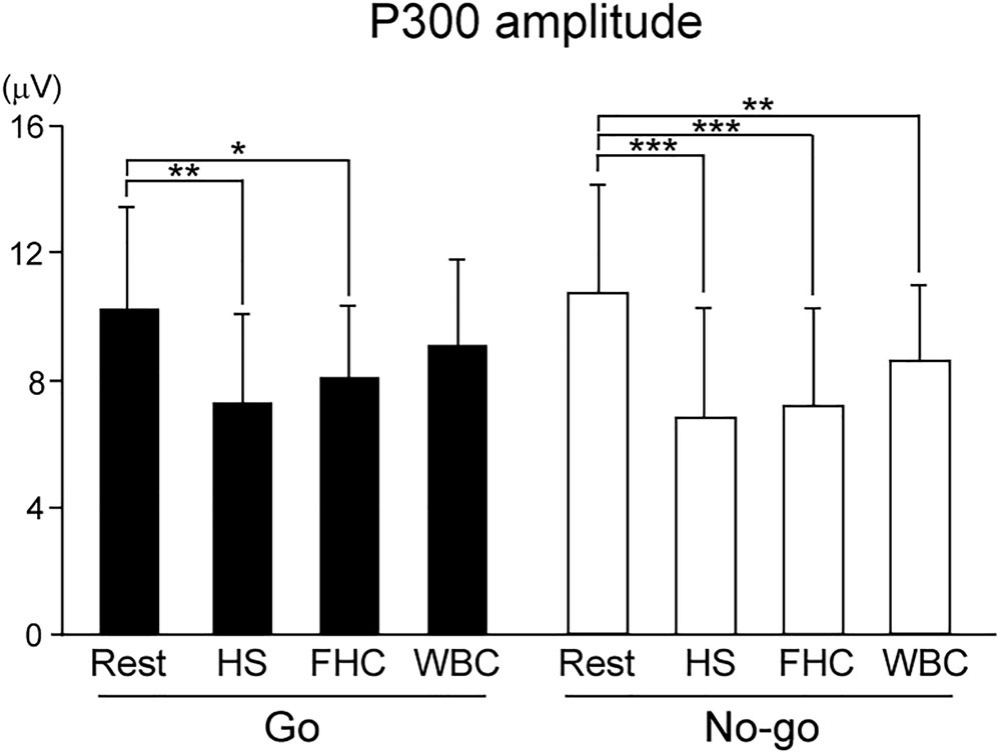
The mean P300 peak amplitude in go/no‐go trials averaged across all electrodes. The amplitude of go‐P300 was significantly reduced during heat stress (HS) and face/head cooling (FHC), but recovered during whole‐body cooling (WBC), whereas the amplitude of no‐go‐P300 was significantly reduced during HS, FHC and WBC. *P < 0.05, **P < 0.01 and ***P < 0.001 from rest. Values are the mean ± SD. Adapted from Shibaskai et al. (2017), with modifications.

Rasmussen et al. (2004) reported that reduced alertness, evaluated by the EEG power spectrum during hyperthermia, was not associated with decreased CBF. Likewise, Schultz Martins et al. (2021) showed that neither skin temperature nor maintenance of the end‐tidal partial pressure of carbon dioxide significantly alter performance on a cognitive test battery, including detection, 2‐back and set‐shifting tasks, during passive hyperthermia. Taken together, these findings indicate that elevated brain temperature itself might contribute to impaired cognitive processing.

Another important consideration during heat stress is that cerebral oxygen delivery comprises both convective and diffusive components. Convective delivery depends on CBF, which transports oxygen to the capillary network, whereas diffusive delivery is determined by tissue oxygen conductivity, capillary geometry and local metabolic rate. As CBF decreases, the oxygen extraction fraction increases to maintain the CMRO_2_. However, when CBF is reduced by ∼50%–60%, this compensatory extraction becomes insufficient to sustain CMRO_2_, defining a critical flow limit. This threshold is further lowered when cerebral metabolism rises; for example, a 10%∼20% increase in metabolism resulting from a 2°C rise in tissue temperature reduces the critical CBF reserve to ∼40%–50% of baseline (Bain et al., 2020, 2015). Elevated CMRO_2_ during hyperthermia might therefore limit the neural resources available for cognitive processing. Nevertheless, Bain et al. (2020) showed that even during severe passive hyperthermia, with a ∼2°C increase in core temperature, CBF decreased by only ∼15%, because oxygen delivery was maintained through a compensatory rise in O_2_ extraction. Thus, in our study, the reduction in CBF was not sufficient to affect oxygen delivery, making it unlikely that inadequate oxygen supply attributable to decreased blood flow had a significant impact.

## 4 HYPOXIC CONDITIONS

High‐altitude exposure induces a variety of physiological changes. In particular, the reduction in the partial pressure of arterial oxygen ($P_{aO_{2}}$) attributable to hypobaric conditions alters respiratory regulation. Respiratory‐induced changes in the partial pressure of arterial carbon dioxide ($P_{\mathrm{aC}O_{2}}$) and $P_{aO_{2}}$ play a major role in regulating CBF. At the onset of hypoxia, stimulation of peripheral chemoreceptors acutely increases ventilation, leading to a reduction in $P_{\mathrm{aC}O_{2}}$. A fall in $P_{\mathrm{aC}O_{2}}$ induces cerebral vasoconstriction and reduces CBF, whereas a fall in $P_{aO_{2}}$ triggers cerebral vasodilatation and increases CBF through cerebrovascular responses. Thus, in hypoxic conditions, CBF is determined by the balance between $P_{aO_{2}}$‐ and $P_{\mathrm{aC}O_{2}}$‐mediated effects through respiration and oxygen delivery to the blood. However, it is important to note that both cerebral vasculature and respiratory responses adapt to hypoxia, causing CBF regulation to vary depending on the duration of exposure (Ainslie & Ogoh, 2010). Against this background, hypoxia associated with high‐altitude exposure or chronic lung disease alters CBF and reduces cerebral oxygen delivery to the anterior cerebral regions. Ogoh et al. (2021) demonstrated that during prolonged hypoxia (fraction of inspired O_2_ = 12%), internal carotid artery blood flow initially increased owing to compensatory vasodilatation and elevated velocity, but this response was insufficient to offset the decrease in arterial oxygen content, resulting in reduced oxygen delivery to the anterior circulation. Consequently, hypoxia affects cerebral metabolism. Zhang et al. (2020) focused not on metabolism per se, but on the altered relationship between CBF and CMRO_2_ during mild hypoxia, demonstrating that neurovascular coupling was attenuated compared with normoxia.

Previous studies suggested that 5000 m can be considered a reference point, above which impairments have been observed across multiple aspects of memory, including learning (Bouquet et al., 1999), spatial memory (Nelson, 1982) and working memory (Champod et al., 2013; Malle et al., 2013; Yan et al., 2011). In addition, a negative correlation between altitude and cognitive function has been reported (Li et al., 2000; Merz et al., 2013; Neuhaus & Hinkelbein, 2014; Rainford & Gradwell, 2006). These findings demonstrate that cognitive function during mountain expeditions can be affected not only by hypobaric hypoxia but also by other high‐altitude‐related factors, such as cold, dehydration (Hayashi et al., 2005) and sleep deficiency (Virués‐Ortega et al., 2004). Heinrich et al. (2019) investigated the effects of hypoxia and sleep quality on cognitive function and mood during high‐altitude exposure and suggested that hypoxia itself is a primary factor contributing to impairments in executive function, emotion recognition and attentional control. However, given that their assessments relied mainly on subjective measures, objective evaluations are needed to substantiate these findings.

Recently, to address this question, we examined the effects of acute hypoxia on higher‐order and somatosensory cognitive processing using ERPs and somatosensory evoked potentials (SEPs), obtained by time‐locked averaging of the EEG with high temporal resolution (Nakata et al., 2017). ERPs reflect higher‐level cognitive processes, including selective attention, expectancy and memory updating, and have thus been widely used to evaluate human higher cognitive function. Neural activity related to motor executive and inhibitory processing can be examined using two ERP components: a negative deflection occurring ∼140–300 ms after stimulus onset (N140) and a positive deflection occurring at ∼300–600 ms (P300), measured in go/no‐go trials, respectively (Nakata et al., 2004, 2015). Consistent with previous studies, middle cerebral artery mean blood velocity (MCA *V*_mean_) decreased during hypoxic inhalation, primarily owing to reductions in $P_{\mathrm{aC}O_{2}}$ resulting from hyperventilation. Interestingly, during hypoxia, MCA *V*_mean_ remained unchanged during the go/no‐go task, whereas a marked increase was observed during the same task in normoxia (Figure 4). Consistent with previous studies, MCA *V*_mean_ decreased during hypoxic inhalation. Interestingly, during hypoxia, MCA *V*_mean_ remained unchanged in the go/no‐go task, whereas a marked increase was observed during the same task during normoxia (Figure 4). Notably, motor executive and inhibitory processing, evaluated by the peak amplitudes of go‐P300 and no‐go‐P300, respectively, were reduced during acute hypoxia (Figure 5). Moreover, go‐P300 latency was also delayed, reflecting slower stimulus classification. In contrast, N140 and SEP amplitudes and latencies were unaffected. In a similar manner to high‐altitude exposure, our recent study demonstrated that acute hypoxia, low oxygen itself (normobaric), impairs cognitive function. Specifically, we confirmed that acute hypoxia disrupts motor executive and inhibitory processing, but not somatosensory processing. However, the physiological mechanisms underlying hypoxia‐induced cognitive impairment remain unclear.

**FIGURE 4:**
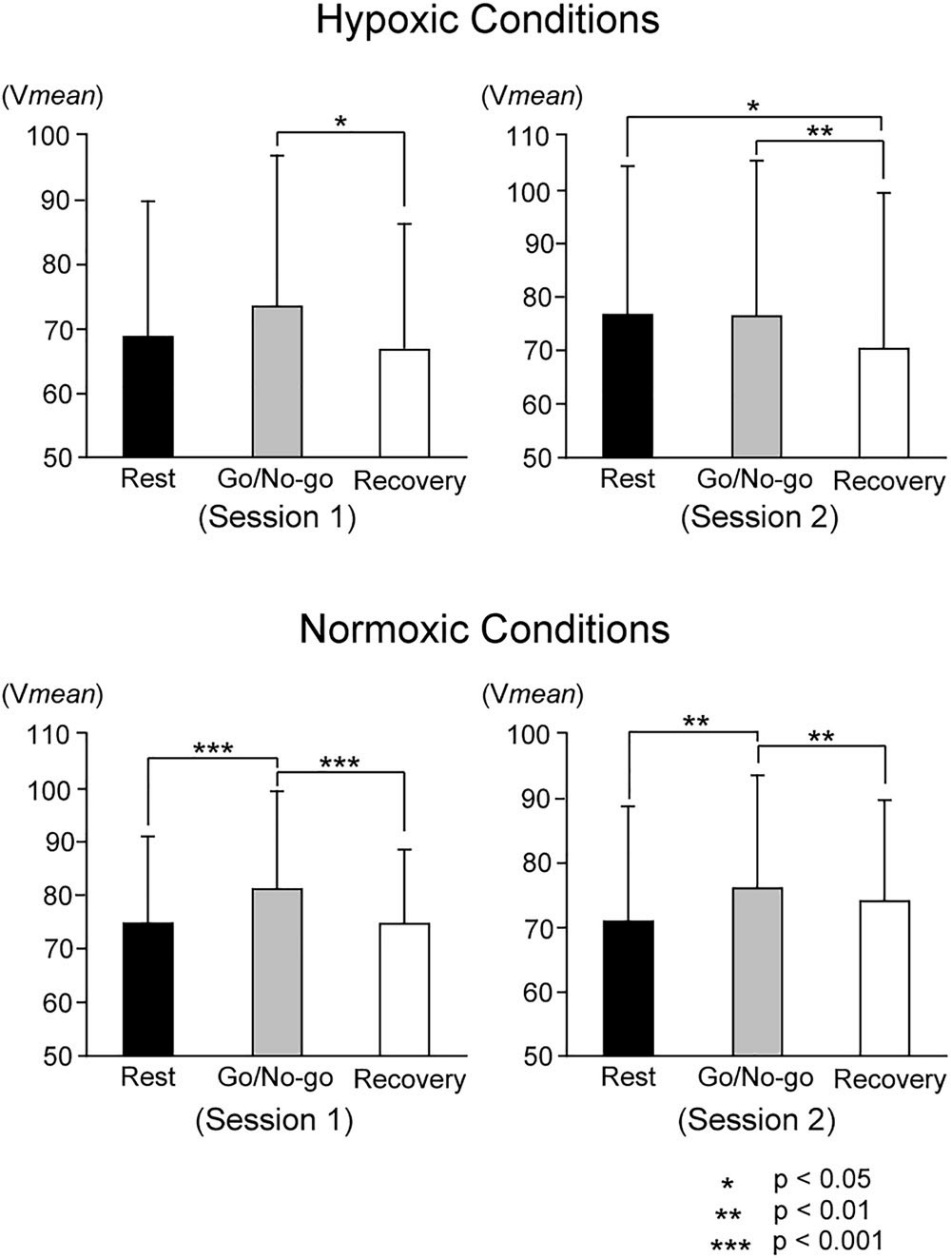
Data on middle cerebral artery mean blood velocity (MCA V_mean_) in hypoxic and normoxic conditions. Values are the mean ± SD. Adapted from Nakata et al. (2017), with modifications.

**FIGURE 5:**
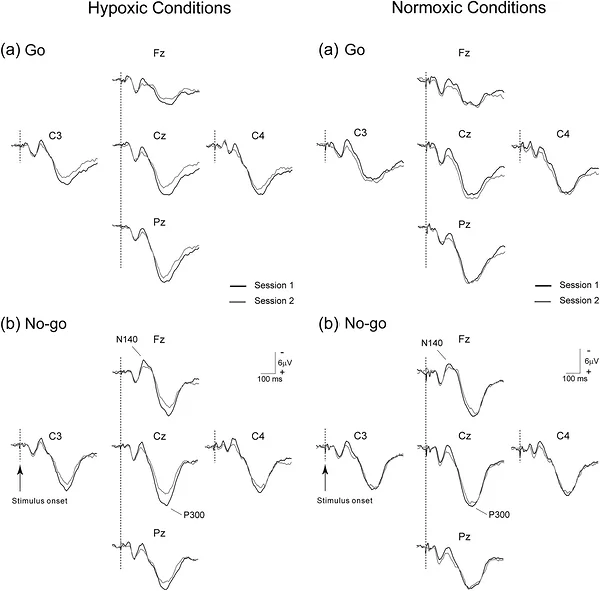
Grand‐averaged event‐related potential (ERP) waveforms at five electrode sites (Fz, Cz, Pz, C3 and C4) during go/no‐go trials in each session. The amplitudes of both go‐P300 and no‐go‐P300 components were markedly reduced in Session 2 in the hypoxic conditions, but not in the normoxic conditions. Reproduced from Nakata et al. (2017).

Alterations in neurotransmitter function, CBF, reduced barometric pressure, $P_{aO_{2}}$ and, in some cases, cerebral metabolism have been recognized as key factors influencing cognitive performance during acute exercise in hypoxic conditions (Ando et al., 2020; McMorris et al., 2017). Given that hypoxia alters CBF via respiratory chemoreflex‐induced hyperventilation (Ogoh, 2015) and that CBF is closely related to $P_{aO_{2}}$ and cerebral metabolism, both of which are key determinants of cognitive function, we hypothesized that an acute hypoxia‐induced decrease in CBF might contribute to cognitive impairment in hypoxic conditions. To test this hypothesis, we investigated the effects of a hypercapnia‐induced increase in CBF and hyperventilation (hypocapnia)‐induced decrease in CBF on cognitive function, assessed using ERPs (Ogoh et al., 2024).

Participants completed four somatosensory go/no‐go tasks sessions in different respiratory conditions: normal breathing (NB); rapid breathing (RB); NB with hypercapnic gas (NB+Gas); and RB with hypercapnic gas (RB+Gas). NB involved ∼15 breaths/min of room air, RB ∼40 breaths/min, NB+Gas ∼15 breaths/min of 5% CO_2_/21% O_2_, and RB+Gas ∼40 breaths/min of the same gas. Hypercapnia increased MCA *V*_mean_, whereas hyperventilation decreased it. Increased MCA *V*_mean_ did not affect the P300 amplitude, whereas decreased MCA *V*_mean_ from hyperventilation attenuated it. Even with combined hypercapnia and hyperventilation, P300 remained reduced, indicating that attenuation was primarily attributable to hyperventilation (dual task) rather than CBF reduction. Overall, alterations in CBF alone did not affect cognitive function during the task, providing important insights into CBF regulation and cerebral homeostasis.

However, it is noteworthy that the duration of hypoxia exposure might influence cognitive function. For example, Davranche et al. (2016) reported that cognitive function was impaired during the early hours of hypoxia exposure, but this impairment disappeared by the second day. This temporal pattern of cognitive changes resembles the adaptation of CBF to hypoxia: acute hypoxia decreases CBF, whereas prolonged exposure increases it, returning to baseline levels by the second day, probably attributable to metabolic compensation and normalization of arterial pH as the ventilatory response stabilizes. These observations suggest that, in hypoxic conditions, alterations in CBF might contribute to changes in cognitive function, in concert with other physiological factors, such as oxygen availability, neural metabolism and neurotransmission. This further suggests that reduced blood oxygen concentration, as occurs in hypoxia, might fall below the critical threshold necessary to sustain cognitive performance. In contrast, with normal blood oxygen levels, physiological fluctuations in CBF are unlikely to fall below this threshold and, therefore, might not pose a risk of cognitive impairment. Although the precise mechanisms remain unclear, hypoxic conditions, including respiratory diseases (such as chronic obstructive pulmonary disease), which impair oxygen uptake, might require careful consideration of CBF regulation as a key factor for maintaining cognitive function and reducing the risk of dementia.

## 5 GENERAL DISCUSSION

The present review showed the relationship between CBF and cognitive function, as assessed by EEG‐ERPs, in two simulated laboratory experiments: hot and hypoxic environments. During exposure to a hot environment, the reduction in CBF associated with elevated body temperature might not necessarily impair cognitive function; however, in hypoxic conditions, the absence of an increase in CBF during cognitive tasks suggests that acute reductions in CBF might adversely affect cognitive function.

As mentioned in the Introduction, several previous studies suggested a positive association between CBF and cognitive performance, particularly in ageing populations and in disease states (Iturria‐Medina et al., 2016; Wolters et al., 2017). Reduced resting CBF has been linked to poorer cognitive function, slower processing speed and increased risk of dementia (de la Torre, 2012). Exercise interventions, which often increase CBF, are reported to improve aspects of cognition acutely (Ishihara et al., 2021; Lambourne & Tomporowski, 2010). These findings support the cerebral perfusion hypothesis of cognitive decline, wherein inadequate cerebral perfusion compromises neural metabolism, thereby impairing functioning. However, it should be acknowledged that the direction of this relationship remains debated. Enhanced neuronal metabolism during exercise, such as increased glucose uptake and mitochondrial activity, might drive elevated CBF via neurovascular coupling, rather than solely reflecting improved perfusion. Thus, exercise‐induced vascular and metabolic adaptations are likely to interact to support cognitive function. However, our findings indicate that acute manipulations of CBF do not necessarily modulate cognitive function, at least in healthy young adults. This agrees with some neuroimaging studies showing that task performance can remain stable despite significant regional CBF reductions, provided that the metabolic demand is met (Ogoh, 2017). It is plausible that cognitive function is robust to short‐term CBF fluctuations owing to compensatory mechanisms, such as an increased oxygen extraction fraction, redistribution of flow to active cortical areas or recruitment of alternative neural networks.

We assessed cognitive processing with ERPs across three different modalities (auditory, somatosensory and visual stimuli) and three different simple cognitive protocols (oddball, go/no‐go and flanker tasks), all of which demonstrated similar reductions in P300 amplitudes during mild heat stress. In the experiment in which CBF was manipulated, whole‐body heating reduced internal carotid arrery (ICA) blood flow, and although ICA blood flow was subsequently restored to the baseline level by cooling the head and face whilst maintaining an elevated core temperature, ERP indices of executive (go‐P300) and inhibitory (no‐go‐P300) processing remained impaired. This dissociation suggests that the observed cognitive deficits were not attributable solely to reduced CBF. The absence of cognitive recovery with restored ICA flow is consistent with the study by Rasmussen et al. (2004), who found that reduced alertness during hyperthermia was not associated with reduced CBF. These findings suggest that factors beyond cerebral perfusion, such as direct thermal effects on neuronal excitability, synaptic transmission or neurotransmitter dynamics, might underlie the persistent cognitive impairments observed during heat stress.

In the hypoxic experiment, the attenuation of both go‐P300 and no‐go amplitudes during hypoxia suggests that the neural networks subserving motor executive and inhibitory control are particularly vulnerable to reduced oxygen availability. Although performing a cognitive task, such as the go/no‐go task, markedly increases CBF (MCA *V*_mean_ in our data) during nomoxia, it does not change during hypoxia. Thus, the reduction in cerebral oxygen delivery might be accentuated further by decreased CBF, potentially affecting cognitive performance. For this, the duration of hypoxic exposure should be taken into account. That is, during acute hypoxia, CBF might decrease initially but then quickly return to the baseline and subsequently increase. In a subsequent experiment, we manipulated respiratory conditions to modulate CBF during cognitive tasks. Hyperventilation reduced CBF and impaired cognitive performance; however, even when hypercapnic gas was added to hyperventilation to restore CBF to baseline levels, cognitive performance remained impaired. Although the influence of the dual task cannot be excluded and requires further investigation, these results suggest that hypoxia‐induced cognitive impairment might not be attributable solely to changes in CBF. In healthy young adults, autoregulatory mechanisms and neurovascular coupling might maintain adequate oxygen and substrate delivery despite marked changes in bulk flow. In contrast, in older adults or those with cerebrovascular pathology, a reduced vascular reserve could make cognition more sensitive to changes in perfusion (Wolters et al., 2017).

Our experiment had several methodological limitations. First, the ERP‐based cognitive assessment focused on motor execution and inhibition in a go/no‐go task. Although this protocol is well suited for probing executive control, it might not capture other cognitive domains, such as working memory or visuospatial processing, which could exhibit different sensitivities to CBF changes. Second, our participants were healthy young adults, limiting generalizability to ageing or clinical populations, in which vascular contributions to cognition might be more marked. Third, both experimental manipulations (heat stress and respiratory gas challenges) were acute and controlled and might not fully represent chronic physiological conditions associated with sustained CBF alterations.

Collectively, studies on heat and hypoxia indicate that the relationship between CBF and cognitive function is highly context dependent. Although increased CBF has been associated with improved cognitive performance in conditions such as ageing and exercise (Ogoh & Ainslie, 2009; Wolters et al., 2017), several experimental studies, including those in heat or hypoxic stress, demonstrate that acute alterations in CBF do not necessarily translate into cognitive improvement (Nakata et al., 2017; Ogoh et al., 2024; Shibasaki et al., 2017). These observations suggest that other factors, such as neural metabolism, neurotransmitter dynamics and thermoregulatory or respiratory adjustments, might play a dominant role in modulating cognition during physiological stress. More broadly, evidence from environmental, ageing and exercise studies highlights that cognitive function is supported by complex neurovascular and neurochemical interactions rather than by CBF alone. Mechanisms such as neurotrophic factor release, improved neural efficiency and enhanced network connectivity are likely to interact with perfusion to sustain cognitive performance (Basso & Suzuki, 2017; McMorris, 2021). Therefore, maintaining optimal cognitive function during stress requires a multifactorial understanding that integrates vascular, metabolic and neural components.

## 6 CONCLUSION

In conclusion, this review examined the link between CBF and cognitive function under passive heat stress and acute hypoxia. Across both conditions, acute changes in CBF did not correspond directly to alterations in cognition, as assessed by ERPs. Instead, cognitive impairments were more closely associated with elevated brain temperature in hyperthermia and reduced oxygen availability in hypoxia. These findings indicate that the CBF–cognition relationship is complex and context dependent, rather than strictly linear.

## AUTHOR CONTRIBUTIONS

This manuscript was drafted by Hiroki Nakata, Shigehiko Ogoh, and Manabu Shibasaki. All authors approved the final version of manuscript and agree to be accountable for all aspects of the work in ensuring that questions related to the accuracy or integrity of any part of the work are appropriately investigated and resolved. All persons designated as authors qualify for authorship, and all those who qualify for authorship are listed.

## CONFLICT OF INTEREST

All authors declare no competing interests.
